# Tsetse Bloodmeal Analyses Incriminate the Common Warthog *Phacochoerus africanus* as an Important Cryptic Host of Animal Trypanosomes in Smallholder Cattle Farming Communities in Shimba Hills, Kenya

**DOI:** 10.3390/pathogens10111501

**Published:** 2021-11-18

**Authors:** Faith I. Ebhodaghe, Michael N. Okal, Shewit Kalayou, Armanda D. S. Bastos, Daniel K. Masiga

**Affiliations:** 1 International Centre of Insect Physiology and Ecology, Nairobi P.O. Box 30772-00100, Kenya; mnyanganga@icipe.org (M.N.O.); skalayou@icipe.org (S.K.); 2 Department of Zoology and Entomology, University of Pretoria, Private Bag X20, Pretoria Hatfield 0083, South Africa; adbastos@zoology.up.ac.za

**Keywords:** Trypanosomiasis, nagana, epidemiology, pathogen, spill-over, reservoir, asymptomatic host, wildlife-livestock interface, Shimba Hills, Kenya

## Abstract

Trypanosomes are endemic and retard cattle health in Shimba Hills, Kenya. Wildlife in the area act as reservoirs of the parasites. However, wild animal species that harbor and expose cattle to tsetse-borne trypanosomes are not well known in Shimba Hills. Using xeno-monitoring surveillance to investigate wild animal reservoirs and sources of trypanosomes in Shimba Hills, we screened 696 trypanosome-infected and uninfected tsetse flies for vertebrate DNA using multiple-gene PCR-High Resolution Melting analysis and amplicon sequencing. Results revealed that tsetse flies fed on 13 mammalian species, preferentially *Phacochoerus africanus* (warthogs) (17.39%, 95% CI: 14.56–20.21) and *Bos taurus* (cattle) (11.35%, 95% CI: 8.99–13.71). Some tsetse flies showed positive cases of bloodmeals from multiple hosts (3.45%, 95% CI: 2.09–4.81), including warthog and cattle (0.57%, 95% CI: 0.01–1.14). Importantly, tsetse flies that took bloodmeals from warthog had significant risk of infections with *Trypanosoma vivax* (5.79%, 95% CI: 1.57–10.00), *T. congolense* (7.44%, 95% CI: 2.70–12.18), and *T. brucei sl* (2.48%, 95% CI: −0.33–5.29). These findings implicate warthogs as important reservoirs of tsetse-borne trypanosomes affecting cattle in Shimba Hills and provide valuable epidemiological insights to underpin the parasites targeted management in *Nagana* vector control programs in the area.

## 1. Introduction

Wildlife are reservoirs of a plethora of pathogens including parasites that are transmitted from wildlife to humans and livestock through habitat sharing or dissemination by haematophagous arthropod-vectors [1]. In sub-Saharan Africa, the tsetse-transmitted trypanosomes responsible for *Nagana* cattle disease and human sleeping sickness are examples of arthropod-borne parasites harbored by asymptomatic wildlife hosts [2,3,4,5]. However, most epidemiological studies on trypanosomes in Africa have focused on the tsetse vectors, human and livestock hosts and only rarely on wildlife reservoirs. In the Serengeti National Park (NP) in Tanzania, Kaare et al. [6] identified trypanosomes in different wild animal species, including warthogs which were the only wildlife shown to harbor *Trypanosoma brucei rhodesiense* the causative agent of acute human sleeping sickness in East Africa. Furthermore, warthogs harbored the widest diversity of animal trypanosomes and were thought to be the source of trypanosomes detected in cattle in the area. These results support suggestions that warthogs are among the wildlife species that contribute to maintaining endemicity and transmission of trypanosome infections in the Luangwa Valley, Zambia [2].

The findings in the Serengeti NP [6] and Luangwa Valley [2] and other wildlife areas in the continent [7,8,9,10] suggest that warthogs may be contributing to trypanosome transmission at the wildlife-livestock interface of the Shimba Hills National Reserve (NR) in Kenya following the wildlife species abundance in the area. Previous epidemiological studies in other wildlife areas in Kenya have identified African buffalo *Syncerus caffer*, giraffe *Giraffa camelopardalis*, African savannah elephants *Loxodonta africana*, and hippopotamus *Hippopotamus amphibious* as the dominant reservoirs of trypanosomes [8,11]. Muturi et al. [8] and Makhulu et al. [11] adopted an alternative strategy to wildlife examination based on xeno-monitoring to characterize animal reservoirs of trypanosomes. Xeno-monitoring, a strategy which explores knowledge of the blood-feeding behaviour of tsetse flies to track animal reservoirs and sources of trypanosomes, provides otherwise inaccessible data on available fauna in and outside local sylvatic ecologies and is particularly convenient because of the difficulty in sampling wildlife directly. Xeno-monitoring also allows investigators to profile wildlife hosts of trypanosomes in real-time in high throughput analysis and over extensive landscapes including hard-to-reach locations in areas where capturing wildlife is both difficult and risky.

As part of a prior epidemiological survey [12], different animal species were described as providing bloodmeals for tsetse flies in Shimba Hills. But the relative contributions of these animals to trypanosome infections in cattle populations in smallholder farming systems in the wildlife-livestock interface remains poorly understood. Moreover, the epidemiological survey by Channumsin et al. [12] was restricted to just two locations (Buffalo Ridge and Zunguluka) and was conducted over a brief sample collection period (about four weeks) thus limiting full understanding of the range of animal bloodmeal hosts of tsetse flies in the area. A clear understanding of wildlife reservoirs of trypanosomes in Shimba Hills [13] will help identify areas where parasites spill-over from wildlife to livestock is highest and where cattle are at greatest risk to trypanosome infections. This would expedite a rational design and efficient implementation of targeted interventions against the tsetse-vectors, thereby alleviating *Nagana*’s adverse effects on cattle health and production and smallholder farmer livelihoods in the area.

In this study, we investigated bloodmeal sources of tsetse flies in Shimba Hills. To visualize the feeding behaviours of tsetse flies, we designed a bipartite interaction network used in epidemiological studies [14,15] to illustrate vector-host relationships and an *UpSet* plot to show frequencies of tsetse bloodmeals on multiple hosts. As tsetse flies have an exclusively haematophagous diet and are exposed to trypanosomes only by feeding on infected animal hosts [3], knowledge of animal species from which the infected vectors obtain nourishment could provide insights into probable sources of infections. We therefore characterized vertebrate DNA in tsetse flies in an attempt to track the animal sources of trypanosomes in samples of the vectors from Shimba Hills using molecular tools to screen the vectors for bloodmeal hosts.

## 2. Results

### 2.1. Animal Bloodmeals in Tsetse Flies

Overall, 50.00% (348/696) (95% CI: 46.28–53.72) of tsetse flies screened for vertebrate DNA harbored animal bloodmeals. The proportion of trapped tsetse flies that had detectable bloodmeals was higher in females [54.80% (251/458) (95% CI: 50.23–59.38)] than males [40.76% (97/238) (95% CI: 34.47–47.04)] [Binomial-Generalized Linear Model (B-GLM]: *p* < 0.05) and in *Glossina pallidipes* [53.42% (281/526) (95% CI: 49.15–57.70)] and *G. austeni* [62.50% (25/40) (95% CI: 46.82–78.18)] than *G. brevipalpis* [32.31% (42/130) (95% CI: 24.16–40.45)] (B-GLM: *p* < 0.05) (Table 1). Proportions of bloodmeal-positive tsetse flies were similar between different age groups and collection sites (B-GLM: *p* > 0.05) (Table 1 and Table 2).

**Figure 1:**
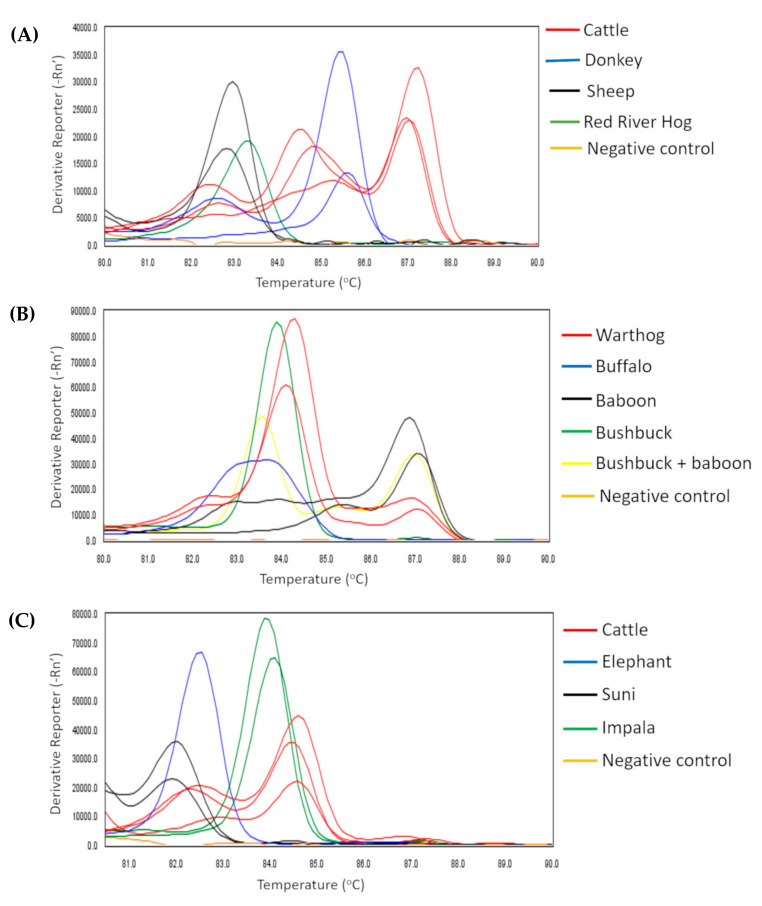

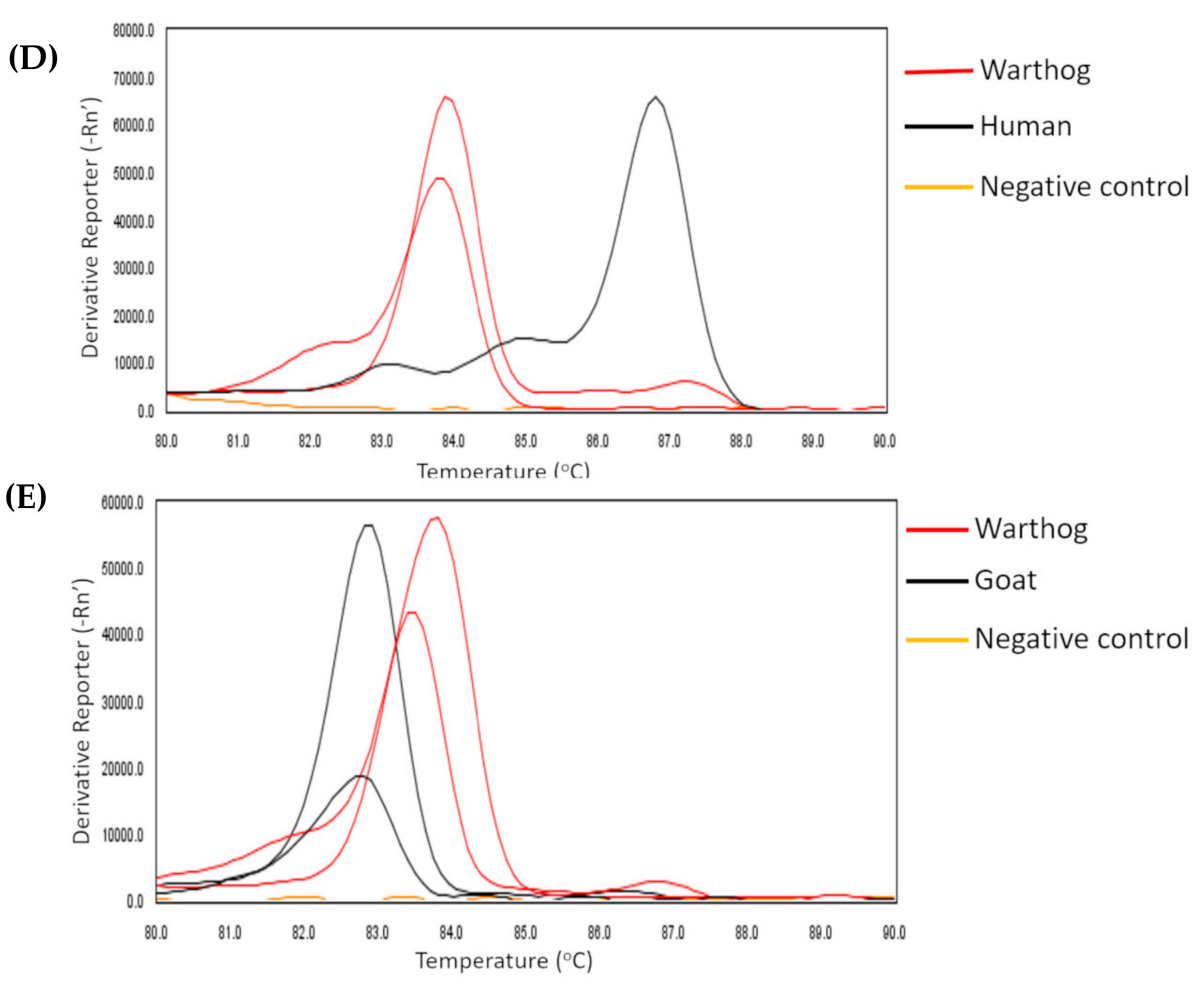
**(A**–**E)** High-Resolution Melt profiles of vertebrate bloodmeals in tsetse flies. Profiles are distinguished using different colours to denote different vertebrate bloodmeal hosts. The identity of a vertebrate bloodmeal is shown on the right side of each graph.

Tsetse flies were positive for bloodmeals of animals from 6 taxonomic families and 13 species (Figure 1, Table 3). These included two suid species *Phacochoerus africanus* (warthog) and *Potamochoerus porcus* (red riverhog) and seven bovid species *Bos taurus* (cattle), *Ovis aries* (sheep), *Syncerus caffer* (buffalo), *Capra hircus* (goat), *Tragelaphus scriptus* (bushbuck), *Neotragus moschatus* (suni) and *Aepyceros melampus* (impala). The other animal bloodmeals identified in tsetse flies were from *Papio anubis* (baboon) (Cercopithecidae), *Loxodonta africana* (elephant) (Elephantidae), *Equus asinus* (donkey) (Equidae) and *Homo sapiens* (human) (Hominidae).

**Figure 2:**
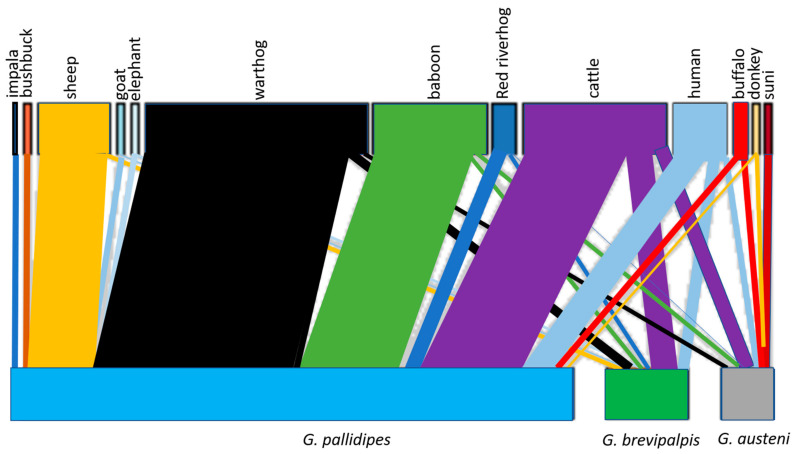
A bipartite network showing interactions between tsetse flies and animal bloodmeal hosts in Shimba Hills, Kenya.

Table 4 shows the number of tsetse fly species that fed on the different animal hosts. This information is visually depicted in the bipartite interaction network in Figure 2. The top and bottom bars on the bipartite network respectively represent animal hosts of tsetse flies and tsetse fly species that fed on these hosts. The size of a bar reflects the number of blood-fed tsetse flies (if it is a bottom bar) or the number of the vector that took bloodmeal from a mammalian species (if it is a top bar). The lines are used to show interactions between tsetse flies and animal bloodmeal hosts. The size of a line is proportional to the number of tsetse flies that took bloodmeals from the mammalian host to which it is connected to. The thick lines between *G. pallidipes* and warthog, cattle, baboon and sheep indicate that the tsetse fly species, more than the other fly species, fed intensely on these animals (Table 4).

*G. pallidipes* being the dominant tsetse flies in Shimba Hills made up 75.57% (526/696) of the total fly individuals screened for vertebrate bloodmeals and thus contributed the highest number of tsetse flies with bloodmeals [80.75% (281/348)]. Over half (59.07%, 166/281) the animal bloodmeals in *G. pallidipes* were from warthog and cattle with 38.43% (108/281) of the fly species bloodmeals from warthog. The proportions of warthog bloodmeals in tsetse flies were similar between blocks though highest in Mlafyeni (Table S1) and significantly different between Mlafyeni and Pengo (B-GLM: *p* < 0.05). Furthermore, tsetse flies in Kinangondogo had the highest rate of cattle bloodmeals but proportions of cattle bloodmeals were insignificantly different between cluster-locations (B-GLM: *p* > 0.05). For all tsetse fly species, B-GLM analyses with pairwise comparisons revealed significantly higher proportion of tsetse flies positive for warthog bloodmeal than other animal bloodmeal (*p* < 0.001) except cattle bloodmeal (*p* > 0.05) (Table 5). Tsetse flies were also more likely to feed on suids (*p* < 0.0001) and bovids (*p* < 0.0001) than other animal hosts (Table 5).

**Figure 3:**
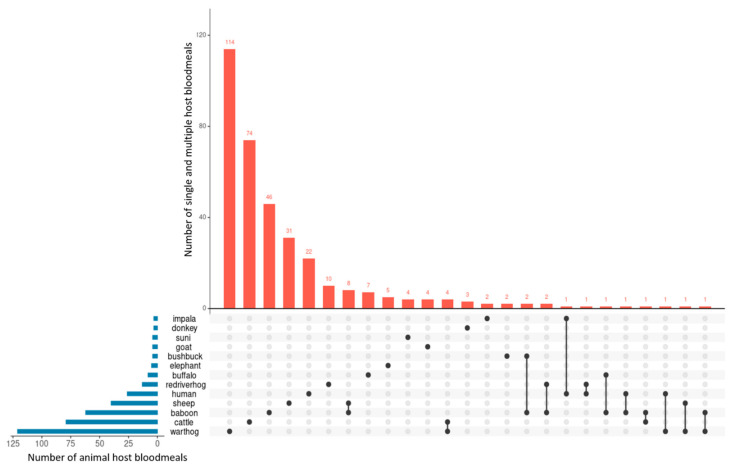
*UpSet* plot showing the frequency of tsetse bloodmeals on single and double animal species in Shimba Hills, Kenya.

The *UpSet* plot in Figure 3 presents the frequency of tsetse bloodmeals on single and double animal species. Warthogs 16.38% (114/696) (95% CI: 13.62–19.14) and cattle 10.63% (74/696) (95% CI: 8.34–12.93) bloodmeals were the most frequently detected in tsetse flies that took bloodmeals from single host species, and baboons plus sheep 1.15% (8/696) (95% CI: 0.36–1.94) and warthogs plus cattle 0.57% (4/696) (95% CI: 0.01–1.14) in the vectors that fed on multiple host species.

### 2.2. Trypanosome Infections in Blood-Fed Tsetse Flies

Overall, 10.92% (38/348) (95% CI: 7.63–14.21) of blood-fed tsetse flies that harbored trypanosome infections had bloodmeals from 10 of the 13 animal species identified. Trypanosomes were not detected in tsetse flies that had fed on impala, goat, and bushbuck.

Trypanosomes in tsetse flies comprised of the livestock pathogens: *T. vivax, T. congolense* Kilifi, *T. congolense* Savannah, *T. simiae* Tsavo, *T. simiae, T. godfreyi* and *T. brucei sl*. Tsetse flies positive for warthog bloodmeals harbored all seven species and subspecies of trypanosomes. Tsetse flies that fed on cattle were positive for all trypanosomes except *T. congolense* Kilifi and *T. brucei sl*. For the other tsetse flies that fed on animal species other than warthogs and cattle, trypanosome infections were comprised of either one or two species but not more.

Tsetse flies that fed on warthogs were significantly exposed to *T. vivax* (Binomial-Generalized Linear Mixed Model [B-GLMM]: *p* < 0.05)*, T. congolense* (B-GLMM: *p* < 0.05) and *T. brucei sl* (B-GLMM: *p* < 0.05) infection risk (Table 6). We also observed significant risk of trypanosome infection (*T. godfreyi*) (B-GLMM: *p* < 0.05) in tsetse flies that took bloodmeals from red riverhog. The only other species of animal bloodmeal outside the Suidae family for which we noted a significant risk of trypanosome infection (*T. congolense*) was suni (Table 6).

## 3. Discussion

Tsetse flies in Shimba Hills fed preferentially on suids (19.25%) and bovids (20.40%). Importantly, the vectors took bloodmeals from mostly warthogs (17.39%) and cattle (11.35%) among the 13 animal species whose bloodmeals we detected in samples of tsetse flies. These findings support previous observations of tsetse preference for cattle bloodmeals [16] and preferential selection of warthogs among wild animals in sylvatic ecologies, including areas where warthogs are sparse in relation to other animal species [17,18,19,20]. In Tanganyika, for example, warthogs made up <3.00% of the total population of wild mammals but >75.00% of the bloodmeals of tsetse flies [19].

Our data show that tsetse flies in Shimba Hills feed preferentially on warthog bloodmeals. However, the underlining reasons for this are not well understood. In a previous study, the mosquito *Anopheles stephensi* preferred to feed on rabbits than guineapigs because the blood from rabbits was of higher nutritional quality and easier to digest [17]. Some experiments confirmed high dietary quality of porcine blood, hence making it the bloodmeal of choice for mass-rearing of tsetse colonies [21]. In one study, it was discovered that warthog skin and urine odours increased catches of tsetse flies [22]. These findings and tsetse disposition for feeding on warthogs in Shimba Hills suggest that further investigation of warthog-based tsetse-attractant semio-chemicals could enhance the toolbox of odour-attractants applied in tsetse surveillance and control in sub-Saharan Africa.

Cattle emit large amounts of tsetse attractant-odours through their urine. This underpins the rationale for urine adoption for tsetse attraction in entomological surveillance and control [23,24,25]. Furthermore, *G. pallidipes* which in Shimba Hills is the dominant tsetse fly species, have an intrinsic predisposition towards bovids, including cattle [16]. It was therefore not surprising that tsetse flies in Shimba Hills were found to have fed frequently on cattle. A contrary finding by Channumsin et al. [12] in Shimba Hills of absence of cattle bloodmeals in tsetse flies may be the result of sampling bias occasioned by the short sampling time (of less than five weeks) reported in that study.

The frequent detection of trypanosomes in tsetse flies positive for warthog and cattle bloodmeals indicates that trypanosomes may move between the sylvatic and domestic cycles. This may explain why high prevalence of trypanosomes in cattle is common in this area, with reports confirming infections in nearly half of cattle livestock assessed during high transmission season [13]. Based on our observations of high diversity and rate of trypanosomes in tsetse flies positive for warthog bloodmeals, it is likely that warthogs play an important role as cryptic reservoirs and epidemiological drivers of cattle trypanosome parasites responsible for *Nagana* disease in smallholder farming systems in the wildlife interface of Shimba Hills.

Tourism, cattle herding, and land cultivation at the Shimba Hills Wildlife Reserve boundary are important factors that expose humans to attacks by tsetse flies. However, we could only detect human DNA in a few tsetse flies, possibly because the savannah tsetse fly species endemic in Shimba Hills are generally averse to feeding on humans [26]. The case is different for riverine tsetse flies, for example *G. fuscipes fuscipes* which feed frequently on humans [16] and in the process transmit *T. b. rhodesiense* responsible for the human sleeping sickness disease in Kenya and other East African countries except for northwest Uganda [4]. In Kenya, sleeping sickness is presently only endemic to the western region bordering Uganda but absent in the coastal area where Shimba Hills is located [27]; Kenya Tsetse and Trypanosomiasis Eradication Council KENTTEC, 2019 www.kenttec.go.ke, assessed on 3 November 2021).

Some animal species were detected infrequently in tsetse fly bloodmeals, probably because of their sparse presence in Shimba Hills. However, infrequent detections of Suni antelope, goat, and impala in Shimba Hills could be explained by the defensive behaviors of these animals against tsetse flies during attempts by the vectors to feed [3]. For sheep, the body covering by thick-wool makes it difficult for tsetse flies to obtain bloodmeals. Elephants have a non-uniform spatial distribution in Shimba Hills being mainly found in areas around Mlafyeni in proximity to the Mwalunganje Elephant Sanctuary [20]. Aside from Mlafyeni and the nearby Pengo and Kizibe, the only other location where we detected elephant bloodmeals in tsetse was in Kinangondogo. Still, the finding was made in a single *G. brevipalpis*, which according to Weitz [16] prefers elephants. This may also be due to the preference of *G. brevipalpis* for forested areas, where elephants in the Shimba Hills National Reserve are frequently found.

Allomonal volatile emissions may explain the absence in tsetse flies of bloodmeals from zebra [28] and waterbuck [13,29], both of which are present in Shimba Hills (Kenya Wildlife Service KWS 2021, www.kws.go.ke/content/shimba-hills-national-reserve, assessed on 3 November 2021). However, skin coloration patterns in zebra are believed to confuse tsetse flies and discourage vector attacks [30,31,32]. Even though we did not detect bloodmeals of zebra, waterbuck, and several other animal species (e.g., giraffe and monitor lizard) previously shown to be fed on by tsetse flies [3], bloodmeal host diversity in tsetse flies was high in Shimba Hills in comparison to reports from some similar ecologies, for example, the Kafue National Park Zambia and Hurungwe Game Reserve Zimbabwe [33]. Large fauna community, extensive spatio-temporal samplings, and adoption of multiple gene-markers to segregate DNA of vertebrates in a high-throughput analysis using the sensitive PCR-HRM technique [34] are important factors which contributed to the wide diversity of tsetse fly bloodmeal hosts in Shimba Hills.

Multiple-host feeding by tsetse flies was presumably the result of certain animals’ anti-feeding behaviours to discourage the vectors from biting attacks. Baboons, like goats and impala, display defensive behaviors against tsetse flies, hence it was not surprising that seven of the twelve sets of multiple hosts involved baboons. Disruption of tsetse-feeding before repletion on a host causes the vectors to switch to other hosts to continue feeding, thus allowing trypanosome-dissemination among and between wildlife and livestock [3,12]. In Shimba Hills, over half the cases of multiple-host feeding involved wildlife and livestock, prominently baboons and sheep, and warthogs and cattle. The finding of warthog and cattle bloodmeals in individual tsetse flies is further evidence that cattle in Shimba Hills are exposed to trypanosomes from warthogs.

Warthog and cattle multiple bloodmeals were detected in *G. pallidipes* and *G. austeni* and in male and female tsetse flies. However, *G. pallidipes* and female tsetse flies have a higher potential for trypanosome transmission in Shimba Hills because they make up >90.00% of the tsetse flies in Shimba Hills, outlive their male counterparts [35,36], and have relatively high rates of displacement which allows them to feed on and distribute infections among a large repertoire of animal species [37]. True to this, the rate of trypanosome infection was higher in older tsetse flies and in female tsetse flies. The wider host range in young flies (data not shown) might just have been because they have a much greater quest for bloodmeals and consequently are more elastic in choice of hosts.

Stationary-baits for tsetse control in Shimba Hills should ideally target *G. pallidipes* because of the fly species high feeding rate on warthogs and cattle with deployment of the control tools prioritized to areas where warthogs are abundant and co-exist with cattle. It might, however, be more effective to integrate stationary-baits with live-baits (or synthetic tsetse repellent odour-treatment of cattle [13]) since tsetse flies in Shimba Hills also feed abundantly on cattle. The live-bait technique in Shimba Hills would have an added advantage of also controlling for ticks, which in the area transmit a large variety of pathogens [38], including *Theileria parva* responsible for the East Coast Fever and which in an epidemiological survey was detected in warthogs in the area [39].

## 4. Materials and Methods

### 4.1. Ethical Consent

The study received ethical consent from Kenya’s National Commission for Science, Technology and Innovation (License No.: NACOSTI/P/20/7344) and the Pwani University Ethics Review (approval number ERC/EXT/002/2020). The study was conducted according to guidelines stipulated by the International Centre of Insect Physiology and Ecology *icipe* Kenya. Collections of tsetse flies were done in collaboration with local communities, the Kenya Tsetse and Trypanosomiasis Eradication Council (KENTTEC), and the Kenya Wildlife Service (KWS).

### 4.2. Study Area

**Figure 4:**
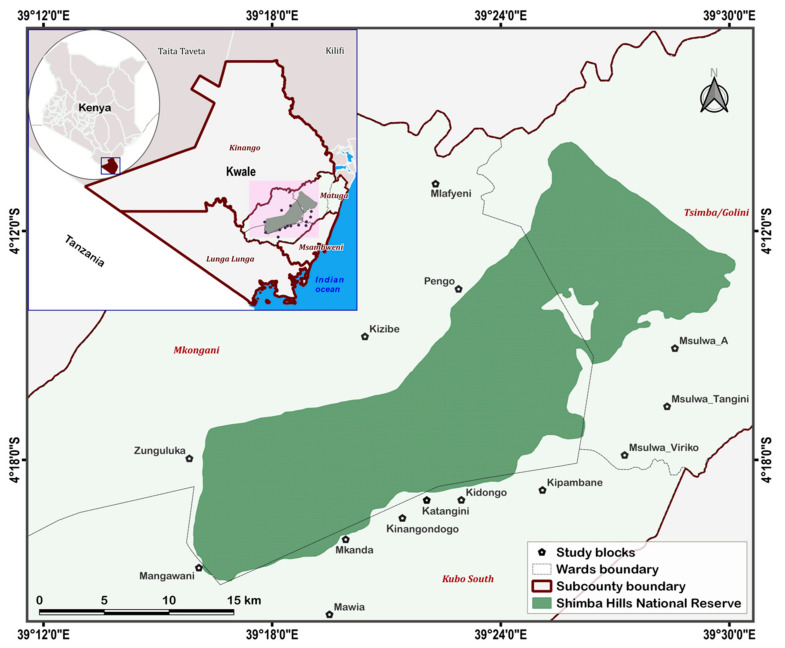
Map of study locations in Shimba Hills in Kwale county, Kenya.

The Shimba Hills NR is located in Kwale County in southeast Kenya (Figure 4). A major wildlife area in East Africa, the Reserve is just ~218 km^2^ yet hosts Kenya’s highest density of elephants (*Loxodonta africana*) [40]. Further, it is home to a wide biodiversity and accommodates important vertebrates including threatened and endangered animal species prominently the Roosevelt’s sable antelope *Hippotragus niger* (Kenya Wildlife Service KWS 2021, www.kws.go.ke, assessed on 3 November 2021). Among animal species domiciled in the area are warthogs (*Phacochoerus africanus*), bushbuck (*Tragelaphus scriptus*), and buffalo (*Syncerus scaffer*). The Shimba Hills area is warm and moist, with average annual temperature and rainfall of ~24 °C and 1150 mm, respectively. The area experiences bimodal rainfall patterns characterized by long rains from March to May, sometimes extending to July, and short rains from October to December. Main economic activities in communities residing at the edge of the reserve are crop and livestock (mainly cattle) production. Vegetation is green year-round hence encouraging intensive cropping activities and discouraging seasonal livestock migration.

### 4.3. Tsetse Fly Collection and Characterization

Samples of tsetse flies were collected over a 10-month period (November 2018 to September 2019) in the Shimba Hills wildlife-livestock interface. Biconical traps for tsetse collection [41] were baited with cow urine and acetone at respective release rates of 1000 mg/h and 500 mg/h and deployed at a density of 1 trap per km^2^ within 5 km of the border of the reserve, over an area stretching ~230 km^2^. Collections of tsetse flies were done bi-monthly throughout the sampling period, across different vegetation landscapes, and in locations at varying proximities to the Shimba Hills NR. Tsetse flies were identified using established taxonomic keys [42], sorted according to sex and species, and subsequently stored in 95% ethanol. Each fly sample was later assessed for age based on the wing fray scoring technique developed by Jackson [43].

### 4.4. Identification of Vertebrate Bloodmeal Sources in Tsetse Flies

Tsetse flies were sterilized in alcohol, air-dried, and crushed using a Mini-Beadbeater-16 (BioSpec, Bartlesville, OK, USA). This was followed by DNA extraction from crushed fly samples using Genomic DNA extraction kits (Bioloine, London, UK) according to the manufacturer’s instructions for animal tissues. Two vertebrate mitochondrial genes were then targeted in separate Polymerase Chain Reactions (PCRs): the first, the *16S* ribosomal RNA gene was amplified with Vert *16S* For: 5′-GAGAAGACCCTRTGGARCTT-3′ and Vert *16S* Rev: 5′-CGCTGTTATCCCTAGGGTA-3′ primers which target a ~200 bp region [44] and the second, the cytochrome *b* gene was amplified with the Cyt *b* For: 5′-CCCCTCAGAATGATATTTGTCCTCA-3′ and Cyt *b* Rev: 5′-CATCCAACATCTCAGCATGATGAAA-3′ primers that target a ~383 bp region [45]. For each PCR-reaction, we used 0.5 μM of each Forward and Reverse primer (Macrogen, Europe, Amsterdam, The Netherlands) in a 10 μL reaction-volume comprising of 1 μL template DNA and 2 μL of pre-formulated 5X HOT FIREPol^®^ EvaGreen^®^ HRM Mix, (Solis BioDyne, Tartu, Estonia). DNA amplifications were carried out for *16S* ribosomal *RNA* and cytochrome *b* in a Rotor-Gene Q thermocycler (Qiagen, Hilden, Germany) and QuantStudio 3 Real-Time PCR System thermal cycler (MicroAmp^®^; Applied Biosystems, Inc., Foster city, CA, USA), respectively with the following thermal cycling conditions: initial denaturation for 15min at 95 °C, followed by 40 cycles of denaturation at 95 °C for 40 s, annealing at 56 °C for 20 s, and extension at 72 °C for 30 s, and a final extension at 72 °C for 5 min. High-Resolution Melting analysis of amplicons followed immediately with gradual melting from 75 °C to 95 °C. Non-template negative controls were included in the experiments to ascertain the success of each run. DNA extracted from cattle, sheep, donkey, giraffe, bushbuck, baboon, impala, hippopotamus, and human were used as positive controls, and tsetse bloodmeal sources were identified by inspecting HRM profile alignments with those of positive controls. Tsetse flies that fed on multiple hosts had HRM curves aligned with more than one positive controls. Melting profiles were analysed in the software Rotor-Gene Q v2.1 and QuantStudioTM Design & Analysis v1.5.1 depending on the machine used for PCR-HRM analysis. Where a profile was different from those of positive control and could not be clearly identified, samples were subjected to *CO1* gene amplification [46] and amplicon sequencing. PCR-reactions targeting a ~750 bp region of the *CO1* gene were carried out in a 15 μL reaction-volume containing 0.5 μM of each Forward and Reverse primer (Macrogen, Europe) (VF1d For: TCTCAACCAACCACAARGAYATYGG; VR1d Rev: TAGACTTCTGGGTGGCCRAARAAYCA) [46], 2 μL template DNA, 3 μL of 5X HOT FIREPol^®^ Blend Master Mix (Solis BioDyne, Tartu, Estonia) with the following cycling conditions: initial denaturation for 15 min at 95 °C, followed by 40 cycles of denaturation at 95 °C for 20 s, annealing at 57 °C for 30 s, and extension at 72 °C for 60 s, followed by a final extension at 72 °C for 7 min. DNA amplification was ascertained by electrophoresis of PCR-products for 30 min in a 1.5% agarose-gel stained with 5 μg/mL ethidium bromide at 120 V. Unincorporated dNTPs and PCR primers were removed from amplicons using Exo-SAP (USB Corporation, Cleveland, OH, USA). Purified amplicons were then submitted for unidirectional Sanger sequencing at Macrogen in Europe.

### 4.5. Molecular Identification of Trypanosomes in Tsetse Flies

Detection of trypanosomes was done using the same crushed homogenates used for bloodmeal analysis. Amplification of trypanosome DNA was performed in a 10 μL reaction-volume comprising of 1 μL DNA template, 5 µL DreamTaq Master Mix (2X) (Thermo Scientific, UK), and 0.5 μL at 10 μM of each Forward and Reverse ITS-1 primers (CF: CCGGAAGTTCACCGATATTG, BR: TTGCTGCGTTCTTCAACGAA) [47]. Cycling conditions for DNA amplification were initial denaturation for 1 min at 95 °C, followed by 35 cycles of denaturation at 95 °C for 30 s, annealing at 60 °C for 20 s, and extension at 72 °C, and a final extension at 72 °C for 7 min. PCR-products were visualized following 1.5% agarose-gel electrophoresis against a molecular weight maker (Gene-Ruler 100 bp DNA ladder, Thermo Scientific, Lithuania) and ethidium bromide staining (5 µg/mL). Where trypanosome infections were present, the parasite species were characterized by the following unique band sizes: *T. vivax* ~250 bp, *T. godfreyi* ~300 bp, *T. simiae* Tsavo ~370 bp, *T. simiae* ~400 bp, *Trypanozoon* (*T. brucei sp*.) ~480 bp, *T. congolense* Kilifi ~620 bp, and *T. congolense* Savannah/Forest ~700 bp [47]. To confirm trypanosome identity, amplicons were cleaned using Exo-SAP (USB Corporation, Cleveland OH) to remove unincorporated dNTPs and PCR primers and thereafter sent for Sanger sequencing of the ITS1gene [47].

### 4.6. Data Analyses

Returned vertebrate DNA sequences were inspected for quality based on their chromatograph profiles and edited in BioEdit *v7.2.5* [48]. Edited sequences were subjected to BLAST analysis for comparison to nucleotide sequences in the NCBI GeneBank-*nr* database (https://blast.ncbi.nlm.nih.gov/Blast.cgi, assessed on 3 November 2021) and a homology cut-off of 98.00% to 100.00% identity was used to infer vertebrate species. The process of trypanosome DNA identification is reported in a parallel work.

Difference in proportions of bloodmeal-positive tsetse flies were tested for significance using the Binomial Generalized Linear Model [49]. *p*-values were significant if <0.05. Where significant differences were present, *Tukey’s Post-Hoc* test was carried out in the ‘*multcomp’ R* package [50] for pairwise comparisons. Next, Binomial Generalized Linear Mixed Model (B-GLMM) analyses with ‘*trap_ID*’ as random factor were implemented in the *GlmmTMB R* package [51,52] to investigate associations between tsetse fly bloodmeals on animal hosts and the vector risk of exposure to trypanosome infections. Furthermore, we designed a bipartite interaction network in the *bipartite R* package [53] to visually depict animal blood-feeding behavior of tsetse fly species in Shimba Hills. Finally, we used an *UpSet* plot to show the number of tsetse flies that fed on particular animal species and the number of tsetse flies containing bloodmeals from one or multiple animal host species.

## Figures and Tables

**Table 1:** Percentage of tsetse with bloodmeals in Shimba Hills according to intrinsic traits and collection sites.

	Number of Tsetse Flies Screened	% Feeding Rate	95% CI
**Fly sex ‡**			
Female	458	54.80 ^a^	50.23–59.38
Male	238	40.76 ^b^	34.47–47.04
**Fly species ‡**			
*G. austeni*	40	62.50 ^b^	46.82–78.18
*G. brevipalpis*	130	32.31 ^a^	24.16–40.45
*G. pallidipes*	526	53.42 ^b^	49.15–57.70
**Fly age †**			
Juvenile	186	51.61 ^a^	44.36–58.86
Old	155	55.48 ^a^	47.57–63.40
Young	355	46.76 ^a^	41.55–51.98
**Landscape †**			
Cultivated field	144	47.92 ^a^	39.66–56.17
Forest	55	50.91 ^a^	37.27–64.55
Fruit-Orchard	110	53.64 ^a^	44.17–63.10
Grassland	161	50.93 ^a^	43.13–58.74
Peridomicilliary	11	54.55 ^a^	19.46–89.63
Shrubs	215	48.37 ^a^	41.64–55.11
**Distance from the SHNR †**			
<1000 m	614	50.65 ^a^	46.69–54.62
1000 to 1999 m	61	44.26 ^a^	31.44–57.09
>2000 m	21	47.62 ^a^	24.32–70.91

‡ Significant (*p* < 0.05); † Insignificant (*p* > 0.05); Letters in superscript have been used to indicate presence or absence of significant differences in pairwise comparisons between the numbers of tsetse flies positive for animal bloodmeals. Significantly different pairs are denoted using different letters while insignificantly different pairs are indicated using same letters.

**Table 2:** Percentage of tsetse with bloodmeals in Shimba Hills according to cluster-location.

	Number of Tsetse Flies Screened	% Feeding Rate	95% CI
**Cluster †**			
Katangini	25	44.00 ^a^	23.09–64.91
Kidongo	12	50.00 ^a^	16.82–83.18
Kinangondogo	17	47.06 ^a^	20.61–73.51
Kipambane	15	53.33 ^a^	24.74–81.93
Kizibe	134	47.76 ^a^	39.19–56.33
Mangawani	36	52.78 ^a^	35.65–69.91
Mawia	36	44.44 ^a^	27.39–61.50
Mkanda	8	12.50 ^a^	−17.06–42.06
Mlafyeni	160	55.00 ^a^	47.21–62.79
Msulwa A	27	66.67 ^a^	47.66–85.67
Msulwa Tangini	2	100.00 ^a^	100.00–100.00
Msulwa Viriko	6	33.33 ^a^	−20.86–87.53
Pengo	196	46.43 ^a^	39.38–53.47
Zunguluka	22	63.64 ^a^	41.81–85.47

† Insignificant (*p* > 0.05). Letters in superscript are all same and indicate absence of significant differences in the number of tsetse flies positive for animal bloodmeals between pairs of cluster-locations.

**Table 3:** Identification of nucleic acid sequences of vertebrate bloodmeals detected in tsetse flies from Shimba Hills.

Sample ID (GenBank Accession No.)	Block	Latitude	Longitude	Fly Species	Fly Sex	Sequence Length (bp)	Closest Match on GenBank (Location)	Aimal Host Species	Sequence Identity (%)
**GP370** (MZ816958)	Mlafyeni	−4.17453	39.39222	*G. pallidipes*	F	667	DQ409327 (Africa)	*Phacochoerus africanus*	99.55
**GP536** (MZ816959)	Mlafyeni	−4.20606	39.40222	*G. pallidipes*	F	595	MN124266 (Kenya)	*Phacochoerus africanus*	100.00
**GP411** (MZ816967)	Pengo	−4.20742	39.37234	*G. pallidipes*	M	607	MN124266 (Kenya)	*Potamochoerus porcus*	99.34
**GB412** (MZ816968)	Pengo	−4.25076	39.36938	*G. brevipalpis*	F	607	MN124266 (Kenya)	*Potamochoerus porcus*	99.01
**GP425** (MZ816969)	Pengo	−4.25076	39.36938	*G. pallidipes*	F	607	MN124266 (Kenya)	*Potamochoerus porcus*	99.38
**GB762** (MZ816966)	Pengo	−4.22782	39.37926	*G. brevipalpis*	F	607	MN124266 (Kenya)	*Potamochoerus porcus*	99.34
**GP362** (MZ816960)	Mlafyeni	−4.25085	39.36904	*G. pallidipes*	F	652	MN124245 (Kenya)	*Bos taurus*	99.85
**GP89** (MZ816962)	Mangawani	−4.3584	39.27996	*G. pallidipes*	F	652	MT576844 (China)	*Bos taurus*	100.00
**GP888** (MZ816961)	Mangawani	−4.3584	39.27996	*G. pallidipes*	F	652	MT576844 (China)	*Bos taurus*	100.00
**GB349** (MZ816970)	kinangondogo	−4.33653	39.34352	*G. brevipalpis*	F	396	MN124271 (Kenya)	*Loxodonta africana*	98.99
**GA379** (MZ816964)	katangini	−4.33402	39.35677	*G. austeni*	F	638	JN645581 (Gabon)	*Neotragus moschatus*	99.84
**GB545** (MZ816965)	Kizibe	−4.27812	39.31002	*G. brevipalpis*	F	538	MF437212 (UAE)	*Homo sapiens*	100.00
**GP665** (MZ816963)	Pengo	−4.28013	39.35485	*G. pallidipes*	F	662	MN124246 (Kenya)	*Capra hircus*	100.00
**GP344** (MZ816971)	Katangini	−4.31766	39.36762	*G. pallidipes*	F	470	MN124256 (Kenya)	*Syncerus caffer*	98.94

Fly sex: M: Male; F: Female.

**Table 4:** Rate of tsetse bloodmeals on animal species according to tsetse fly species.

	*G. austeni* (*n* = 40) †			*G. brevipalpis* (*n* = 130) †			*G. pallidipes* (*n* = 526) ‡		
	No	%	95% CI	No	%	95% CI	No	%	95% CI
Baboon	4	10 ^a^	0.28–19.72	4	3.08 ^a^	0.07–6.09	54	10.27 ^c^	7.66–12.87
Buffalo	2	5 ^a^	−2.06–12.06	0	0	NA	6	1.14 ^a^	0.23–2.05
Bushbuck	0	0	NA	0	0	NA	4	0.76 ^a^	0.02–1.51
Cattle	7	17.5 ^a^	5.19–21.81	14	10.77 ^a^	5.37–16.17	58	11.03 ^c^	8.34–13.71
Donkey	1	2.5 ^a^	−2.56–7.56	0	0	NA	2	0.38 ^a^	−0.15–0.91
Elephant	0	0	NA	3	2.31 ^a^	−0.31–4.92	2	0.38 ^a^	−0.15–0.91
Goat	0	0	NA	1	0.77 ^a^	−0.75–2.29	3	0.57 ^a^	−0.08–1.22
Human	3	7.5 ^a^	−1.03–16.03	6	4.62 ^a^	0.96–8.27	17	3.23 ^ab^	1.72–4.75
Impala	0	0	NA	0	0	NA	3	0.57 ^a^	−0.08–1.22
Red Riverhog	1	2.5 ^a^	−2.56–7.56	4	3.08 ^a^	0.07–6.09	8	1.52 ^a^	0.47–2.57
Sheep	0	0	NA	5	6.65 ^a^	0.50–7.20	35	6.65 ^bc^	4.52–8.79
Suni	4	10 ^a^	0.28–19.72	0	0	NA	0	0	NA
Warthog	6	15 ^a^	3.43–26.57	7	5.38 ^a^	1.45–9.32	108	20.53 ^d^	17.07–23.10

NA: Not Available. ‡ Significant (*p* < 0.05); † Insignificant (*p* > 0.05). Letters in superscript have been used to indicate presence or absence of significant difference in pairwise comparisons between animal hosts regarding the numbers of tsetse flies that fed on them. Significantly different pairs are denoted using different letters while insignificantly different pairs are indicated using same letters.

**Table 5:** Rate of tsetse bloodmeals on animal hosts according to animal family and species.

Host Family	No. of Tsetse Flies	Feeding Rate (%)	95% CI
Bovidae	142	20.40 ^a^	17.40–23.40
Suidae	134	19.25 ^a^	16.32–22.19
Cercopithecidae	62	8.91 ^b^	6.79–11.03
Hominidae	26	3.74 ^c^	2.32–5.15
Elephantidae	5	0.72 ^d^	0.09–1.35
Equidae	3	0.43 ^d^	−0.06–0.92
**Host Species**			
Warthog	121	17.39 ^f^	14.56–20.21
Cattle	79	11.35 ^ef^	8.99–13.71
Baboon	62	8.91 ^de^	6.79–11.03
Sheep	40	5.75 ^cd^	4.01–7.48
Human	26	3.74 ^bc^	2.32–5.15
Red River Hog	13	1.87 ^ab^	0.86–2.88
Buffalo	8	1.15 ^ab^	0.36–1.94
Elephant	5	0.72 ^a^	0.09–1.35
Bushbuck	4	0.57 ^a^	0.01–1.14
Goat	4	0.57 ^a^	0.01–1.14
Suni	4	0.57 ^a^	0.01–1.14
Donkey	3	0.43 ^a^	−0.06–0.92
Impala	3	0.43 ^a^	−0.06–0.92

Letters in superscript have been used to indicate presence or absence of significant difference in pairwise comparisons between animal hosts in the numbers of tsetse flies that fed on them. Significantly different pairs are denoted using different letters while insignificantly different pairs are indicated using same letters.

**Table 6:** Risk of trypanosome infection in tsetse flies that obtained bloodmeals from different animal species.

	Bloodmeal-Positive Tsetse Flies	*T. vivax*		*T. simiae* Tsavo		*T. simiae*	
		% (95% CI)	*p*-Value	% (95% CI)	*p*-Value	% (95% CI)	*p*-Value
Bovidae	142	2.82 (0.06–5.57)	0.746	1.41 (−0.55–3.37)	0.844	0.70 (−0.69–2.10)	0.982
Suidae **‡**	134	5.22 (1.41–9.04)	**0.027**	1.49 (−0.59–3.57)	0.479	1.49 (−0.59–3.57)	0.259
Cercopithecidae	62	1.61 (−1.61–4.84)	0.660	1.61 (−1.61–4.84)	0.886	NA	-
Hominidae	26	3.85 (−4.08–11.77)	0.640	3.85 (−4.08–11.77)	0.093	NA	-
Elephantidae	5	NA	-	NA	-	NA	**-**
Equidae	3	NA	-	NA	-	NA	-
Warthog **‡**	121	5.79 (1.57–10.00)	**0.014**	0.83 (−0.81–2.46)	0.865	1.65 (−0.65–3.96)	0.205
Cattle	79	1.27 (−1.25–3.79)	0.482	2.53 (−1.01–6.07)	0.313	1.27 (−1.25–3.79)	0.548
Baboon	62	1.61 (−1.61–4.84)	0.660	1.61 (−1.61–4.84)	0.886	NA	-
Sheep	40	5.00 (−2.06–12.06)	0.294	NA	-	NA	-
Human	26	3.85 (−4.08–11.77)	0.640	3.85 (−4.08–11.77)	0.093	NA	-
Red River Hog	13	NA	-	7.69 (−9.07–9.07)	0.084	NA	-
Buffalo	8	12.50 (−17.06–42.06)	0.103	NA	-	NA	-
Elephant	5	NA	-	NA	-	NA	**-**
Bushbuck	4	NA	-	NA	-	NA	-
Goat	4	NA	-	NA	-	NA	-
Suni	4	NA	-	NA	-	NA	-
Donkey	3	NA	-	NA	-	NA	-
Impala	3	NA	-	NA	-	NA	-
	**Bloodmeal-Positive Tsetse Flies**	***T. godfreyi***		***T. congolense⁋***		***T. brucei sl***	
		**% (95% CI)**	***p*- ** **Value**	**% (95% CI)**	***p*- ** **Value**	**% (95% CI)**	***p*- ** **Value**
Bovidae	142	0.70 (−0.69–2.10)	0.689	4.93 (−1.33–8.53)	0.345	NA	-
Suidae **‡**	134	1.49 (−0.59–3.57)	0.535	6.72 (−2.42–11.01)	0.060	2.24 (−0.30–4.78)	**0.043**
Cercopithecidae	62	NA	-	NA	-	NA	-
Hominidae	26	NA	-	NA	-	NA	-
Elephantidae	5	NA	-	NA	-	NA	-
Equidae	3	NA	-	NA	-	NA	-
Warthog **‡**	121	0.83 (−0.81–2.46)	0.828	7.44 (2.70–12.18)	**0.033**	2.48 (−0.33–5.29)	**0.031**
Cattle	79	1.27 (−1.25–3.79)	0.806	3.80 (−0.51–8.11)	0.994	NA	-
Baboon	62	NA	-	NA	-	NA	-
Sheep	40	NA	-	2.50 (−2.56–7.56)	0.785	NA	-
Human	26	NA	-	NA	-	NA	-
Red River Hog **‡**	13	7.69 (−9.07–9.07)	**0.046**	NA	-	NA	-
Buffalo	8	NA	-	NA	-	NA	-
Elephant	5	NA	-	NA	-	NA	-
Bushbuck	4	NA	-	NA	-	NA	-
Goat	4	NA	-	NA	-	NA	-
Suni **‡**	4	NA	-	75.00 (4.56–154.56)	**0.0004**	NA	-
Donkey	3	NA	-	NA	-	NA	-
Impala	3	NA	-	NA	-	NA	-

*T. congolense* comprising of both the Kilifi and the Savannah strains. ‡ Significant (*p* < 0.05). NA: Small sample size, or too few number or absence of infection cases.

## Data Availability

The dataset used and/or analysed during the current study are available from the corresponding author FIE on reasonable request. DNA sequences of vertebrate species generated during the current study are available in the GenBank under accession numbers: MZ816958-MZ816971.

## Supplementary Materials

The following are available online at https://www.mdpi.com/article/10.3390/pathogens10111501/s1, Table S1: Data on tsetse fly bloodmeal hosts and trypanosome infections in the different study-blocks in Shimba Hills, Kenya.
