# Supplementary material for: Tsetse Bloodmeal Analyses Incriminate the Common Warthog Phacochoerus africanus as an Important Cryptic Host of Animal Trypanosomes in Smallholder Cattle Farming Communities in Shimba Hills, Kenya
Source: Pathogens. 2021 Nov 18;10(11):1501. doi: 10.3390/pathogens10111501 (PMC8623152; doi:10.3390/pathogens10111501)
Supplement: Supplementary file 1 [file pathogens-10-01501-s001.zip › pathogens-1427467-supplementary.pdf]

Table S1. Data on tsetse fly bloodmeal hosts and trypanosome infections in the different study-blocks in Shimba Hills, Kenya.

**Block**	**Msulwa A**	**Katangini**	**Kidongo**	**Kinagodongo**	**Kipambane**	***Kizibe**	**Mangawani**	**Mawia**	**Mkanda**	***Mlafyeni**	**Pengo**	**Msulwa Tangini**	**Msulwa Viriko**	**Zunguluka**	**No. (%) trypanosome positive flies**
**Latitude**	-4.26385	-4.32020	-4.31760	-4.32486	-4.31628	-4.27156	-4.34504	-4.34638	-4.33767	-4.19133	-4.22542	-4.28598	-4.31175	-4.30689	
**Longitude**	39.46818	39.36612	39.38276	39.35194	39.41810	39.33925	39.26604	39.30693	39.32394	39.39329	39.38154	39.44956	39.43014	39.26701	
**No. of tsetse flies examined**	27	25	12	17	15	134	36	36	8	160	196	2	6	22	696
**No. (%) of blood-fed tsetse flies**	18(66.67)	11(44.00)	6(50.00)	8(47.06)	8(53.33)	64(47.76)	19(52.78)	16(44.44)	1(12.5)	88(55)	91(46.43)	2(100)	2(33.33)	14(63.64)	348 (50.00)
**No. (%) of tsetse fly individuals positive for both wildlife & livestock feeds**	0(0.00)	0(0.00)	0(0.00)	0(0.00)	1(12.50)	3(4.69)	0(0.00)	1(6.25)	0(0.00)	3(3.41)	5(5.49)	0(0.00)	1(50.00)	0(0.00)	14 (2.01)
**Blood meal hosts [No (%)]**															
Baboon (n=62)	2(11.11)	4(36.36)	2(28.57)	0(0.00)	2(22.22)	7(10.61)	1(5.00)	1(5.88)	0(0.00)	14(15.05)	19(19.00)	0(0.00)	0(0.00)	10(62.50)	2(3.22)
Cattle (n=79)	7(38.89)	2(18.18)	0(0.00)	4(50.00)	3(33.33)	19(28.79)	7(35.00)	4(23.53)	0(0.00)	14(15.05)	17(17.00)	0(0.00)	1(33.33)	1(6.25)	6 (7.59)
Warthog (n=121)	7(38.89)	2(18.18)	1(14.29)	1(12.50)	1(11.11)	27(40.91)	5(25.00)	5(29.41)	1(100.00)	47(53.41)	19(19.00)	1(50.00)	1(33.33)	3(18.75)	19 (15.70)
Sheep (n=40)	2(11.11)	0(0.00)	2(28.57)	1(12.50)	1(11.11)	4(6.06)	1(5.00)	2(11.77)	0(0.00)	6(6.45)	19(19.00)	0(0.00)	1(33.33)	1(6.25)	3 (7.50)
Buffalo (n=8)	0(0.00)	1(9.09)	0(0.00)	1(12.50)	1(11.11)	1(1.52)	1(5.00)	1(5.88)	0(0.00)	0(0.00)	1(1.00)	1(50.00)	0(0.00)	0(0.00)	1 (12.50)
Human (n=26)	0(0.00)	1(9.09)	1(14.29)	0(0.00)	1(11.11)	2(3.03)	5(25.00)	2(11.77)	0(0.00)	7(7.53)	7(7.69)	0(0.00)	0(0.00)	0(0.00)	2 (7.69)
Suni (n=4)	0(0.00)	1(9.09)	0(0.00)	0(0.00)	0(0.00)	2(3.03)	0(0.00)	0(0.00)	0(0.00)	0(0.00)	1(1.00)	0(0.00)	0(0.00)	0(0.00)	3 (75.00)
red river hog (n=13)	0(0.00)	0(0.00)	1(14.29)	0(0.00)	0(0.00)	1(1.52)	0(0.00)	0(0.00)	0(0.00)	0(0.00)	11(11.00)	0(0.00)	0(0.00)	0(0.00)	2 (15.38)
Elephant (n=5)	0(0.00)	0(0.00)	0(0.00)	1(12.50)	0(0.00)	1(1.52)	0(0.00)	0(0.00)	0(0.00)	1(1.08)	2(2.00)	0(0.00)	0(0.00)	0(0.00)	1 (20.00)
Donkey (n=3)	0(0.00)	0(0.00)	0(0.00)	0(0.00)	0(0.00)	3(4.55)	0(0.00)	0(0.00)	0(0.00)	0(0.00)	0(0.00)	0(0.00)	0(0.00)	0(0.00)	1 (33.33)
Bushbuck (n=4)	0(0.00)	0(0.00)	0(0.00)	0(0.00)	0(0.00)	0(0.00)	0(0.00)	1(5.88)	0(0.00)	2(2.15)	0(0.00)	0(0.00)	0(0.00)	1(6.25)	0
Impala (n=3)	0(0.00)	0(0.00)	0(0.00)	0(0.00)	0(0.00)	0(0.00)	0(0.00)	1(5.88)	0(0.00)	2(2.15)	0(0.00)	0(0.00)	0(0.00)	0(0.00)	0
Goat (n=4)	0(0.00)	0(0.00)	0(0.00)	0(0.00)	0(0.00)	0(0.00)	0(0.00)	0(0.00)	0(0.00)	0(0.00)	4(4.00)	0(0.00)	0(0.00)	0(0.00)	0
Nil	9	14	6	9	7	70	17	20	7	72	105	0	4	8	348
**No. (%) of trypanosome positive flies**	5(18.52)	2(8.00)	0	3 (17.65)	3 (20.00)	18 (13.43)	1 (2.78)	2 (5.56)	0	13 (8.13)	9 (4.59)	0	2 (33.33)	2 (9.09)	60 (8.62)

*warthog and cattle multiple-feeds in tsetse flies.

Cells highlighted in green to indicate blocks where tsetse flies had fed on multiple-hosts comprising of wildlife and livestock.

Cells highlighted in red to indicate blocks where tsetse flies were positive for trypanosome infections.
